# Second-Line Antiretroviral Therapy in a Workplace and Community-Based Treatment Programme in South Africa: Determinants of Virological Outcome

**DOI:** 10.1371/journal.pone.0036997

**Published:** 2012-05-29

**Authors:** Victoria Johnston, Katherine Fielding, Salome Charalambous, Mildred Mampho, Gavin Churchyard, Andrew Phillips, Alison D. Grant

**Affiliations:** 1 Department of Clinical Research, London School of Hygiene and Tropical Medicine, London, United Kingdom; 2 Department of Infectious Diseases Epidemiology, London School of Hygiene and Tropical Medicine, London, United Kingdom; 3 Department of Research, The Aurum Institute, Johannesburg, South Africa; 4 Centre for the AIDS Programme of Research in South Africa, University of KwaZulu Natal, Durban, South Africa; 5 Department of Infection and Population Health, University College London, London, United Kingdom; Boston University, United States of America

## Abstract

***Background:*** As antiretroviral treatment (ART) programmes in resource-limited settings mature, more patients are experiencing virological failure. Without resistance testing, deciding who should switch to second-line ART can be difficult. The consequences for second-line outcomes are unclear. In a workplace- and community-based multi-site programme, with 6-monthly virological monitoring, we describe outcomes and predictors of viral suppression on second-line, protease inhibitor-based ART.

***Methods:*** We used prospectively collected clinic data from patients commencing first-line ART between 1/1/03 and 31/12/08 to construct a study cohort of patients switched to second-line ART in the presence of a viral load (VL) ≥400 copies/ml. Predictors of VL<400 copies/ml within 15 months of switch were assessed using modified Poisson regression to estimate risk ratios.

***Results:*** 205 workplace patients (91.7% male; median age 43 yrs) and 212 community patients (38.7% male; median age 36 yrs) switched regimens. At switch compared to community patients, workplace patients had a longer duration of viraemia, higher VL, lower CD4 count, and higher reported non-adherence on first-line ART. Non-adherence was the reported reason for switching in a higher proportion of workplace patients. Following switch, 48.3% (workplace) and 72.0% (community) achieved VL<400, with non-adherence (17.9% vs. 1.4%) and virological rebound (35.6% vs. 13.2% with available measures) reported more commonly in the workplace programme. In adjusted analysis of the workplace programme, lower switch VL and younger age were associated with VL<400. In the community programme, shorter duration of viraemia, higher CD4 count and transfers into programme on ART were associated with VL<400.

***Conclusion:*** High levels of viral suppression on second-line ART can be, but are not always, achieved in multi-site treatment programmes with both individual- and programme-level factors influencing outcomes. Strategies to support both healthcare workers and patients during this switch period need to be evaluated; sub-optimal adherence, particularly in the workplace programme must be addressed.

## Introduction

As antiretroviral treatment (ART) programmes in resource-limited settings mature, patients are increasingly experiencing first-line, non-nucleoside reverse transcriptase inhibitor (NNRTI)-based, treatment failure necessitating a switch to second-line, protease inhibitor (PI)-based regimens [1]-[3]. Current rates of switching are low [4]-[5]; by the end of 2010 only 3% of patients in resource-limited settings (excluding South and Central Americas) had switched to second-line ART [1]. Low sensitivity of clinico-immunological definitions of treatment failure are partly responsible for low rates of switching. However programmes, such as those in South Africa which use virological monitoring, also report delays [4]. The reasons are likely to include lack of access to resistance tests to guide decisions, difficulties in excluding non-adherence as a cause of virological failure, and potentially concerns regarding cost and limited availability of subsequent regimens [6]-[7]. In the absence of resistance tests, deciding who has virological failure secondary to resistance is difficult. Studies from programmes which use routine virological monitoring have reported that the proportion of patients with no major drug resistance mutations is 9-60% on first raised viral load (300-1000 copies/ml) [8]-[12], 6-33% at confirmatory raised viral load (300-5000 copies/ml) [10], [13]-[15] and 12% at time of switching to second-line ART [16]; suggesting non-adherence is a major cause of viraemia at these time-points. Switching patients with no detectable resistance to second-line ART is arguably unnecessary, and potentially fails to address the underlying adherence issues. With limited regimen availability, unnecessary switching may compromise future treatment options for the individual, and drive up programme costs. In South Africa second-line ART is estimated to be 2.4 times more expensive per year in care than first-line ART [17].

The consequences of remaining on a virologically-failing first-line regimen include immunological and clinical progression and, with increasing duration of viraemia, accumulation of resistance [18]-[24]. For patients who eventually start second-line ART, the consequences of a switch strategy based on virological monitoring without resistance tests, on subsequent outcomes have not been fully described. Early reports of second-line outcomes appear promising with 78-87% of patients in care 12 months following switch, and 77-85% of those achieving viral suppression [25]-[27]. However, these reports are largely from academic or referral clinics, and it is unclear if the same outcomes will be seen under multi-site programmatic conditions.

This study aimed to describe second-line ART outcomes in a large workplace- and community-based multi-site programme, where, in line with South African national guidelines, 6-monthly viral load (VL) monitoring is standard of care. In addition we assessed whether co-variates available at the time of switch predict early viral suppression on second-line ART.

## Methods

### Study Design and Setting

This observational retrospective cohort analysis used prospectively-collected routine clinical data from the ART programmes of Aurum Institute, South Africa. These programmes, located within five provinces of South Africa (Gauteng, Free State, Limpopo, Mpumalanga and North West), comprise a workplace programme, with 56 clinics serving employees of predominantly mining companies; and a community programme, with 81 urban and peri-urban private general practitioner and non-government organization clinics serving patients with limited resources [28]-[29].

In the workplace, patients were eligible for ART (efavirenz [EFV] or nevirapine [NVP] with zidovudine [AZT], lamivudine [3TC] until 2008, then tenofovir/emtricitabine thereafter) if WHO stage IV, CD4≤250 cells/mm^3^, or CD4≤350 cells/mm^3^ *plus* WHO stage III. In the community programme, criteria for first-line ART (stavudine [d4T], 3TC, and NVP or EFV) were WHO stage IV or CD4≤200 cells/mm^3^. Similar criteria for switching to second-line ART were used in both programmes. Interventions to improve adherence were instigated following the first detectable VL, and VL was repeated 3-6 months later. A switch to second-line ART was recommended in patients with two raised VLs >1000-5000 in the presence of good adherence. Second-line ART comprised AZT, didanosine (ddI) and boosted lopinavir (bLPV); or abacavir (ABC), ddI, bLPV in the community and workplace programmes, respectively. Patients collected ART at 1-3 monthly intervals. All HIV-related treatment was free of charge.

CD4 count and VL were monitored at baseline, 6 weeks and 6 monthly intervals after commencing or switching ART. All community clinics were doctor-led; however some workplace clinics were nurse-led with doctors consulted for management of virological failure. Patients were offered adherence counseling at each attendance, with intensified counseling for those with suboptimal adherence.

### Study Population

Patients were eligible for inclusion in the study if they (1) switched from first- to second-line ART between 1/1/2003 and 31/12/2008; (2) ≥15 years old at switch; and (3) VL >400 copies/ml at switch (regardless of whether criteria for switching, as per programme guidelines, were fulfilled). Data up to 31/3/2010 were included, allowing all patients 15 months potential follow-up.

### Data Collection

At each visit, healthcare workers recorded data on symptoms, self-reported adherence, adverse events, prescriptions and reason for stopping or changing medication on standardized data collection forms. Before commencing ART, data were collected on patient’s self-reported previous exposure to ART. Reasons for leaving the programme, derived from patient or relative self-report, and active follow-up of patients missing appointments, were recorded on deregistration forms. Data capturers entered all forms into a central database with laboratory data transferred electronically from the central laboratory. Where civil identification numbers were available, deaths were identified through the National death register; and in the workplace, through employment records and hospital death registers. Where data were missing, clinic files were reviewed using a standardised data collection form. All community sites used a central off-site pharmacy. These dispensing records were used to confirm regimens and dates dispensed.

### Outcomes

The primary outcome was viral suppression on second-line ART, which was defined as ever having achieved a VL<400 copies/ml between 2 weeks to 15 months of switching regimens. Secondary outcomes were defined as (1) alive and in care: no record of deregistration or loss to follow-up (no clinic contact for ≥6 months) by 15 months; (2) change in CD4 count: CD4 at 12 months (+/−3 months) minus CD4 count at switch (6 months before to 2 weeks after switch); (3) reported non-adherence: patient report of missing any second-line ART based on 7 day recall and/or healthcare worker recorded treatment interruption for non-adherence within 15 months of switch.

### Risk Factors

Exposures on first-line ART (transfers into programme on ART, viral suppression, non-adherence), exposures at time of switch (duration and magnitude of viraemia, CD4 count, reason for switch, calendar year, number of new NRTIs in switch regimen) and demographic data (age, sex, programme) were considered as potential predictors of early virological suppression on second-line ART. An association between adherence on second-line ART and viral suppression on second-line ART was explored, however this variable was not included in our multivariable analysis as it was considered to lie on the causal pathway between our exposures of interest and the primary outcome.

Non-adherence on first-line ART was defined as patient report of missing any first-line ART based on 7 day recall and/or healthcare worker recorded treatment interruption for non-adherence at any time-point on first-line ART. Duration of viraemia was defined as the time period between the first VL >400 copies/ml following viral suppression to date of switch, where all interim VLs were >400 copies/ml. For patients with more than one episode of viraemia and re-suppression on first-line ART, only the viraemic period immediately preceding switch was considered. The variable was categorised as<12 months and ≥12 months. Not all patients were known to have achieved viral suppression on first-line ART therefore the following assumptions were made: (1) ART-naive patients with no evidence of viral suppression on first-line ART were considered viraemic since initiating ART; (2) patients who were transferred in with no subsequent viral suppression on first-line ART were categorised as viraemic for ≥12months.

Healthcare workers could document more than one reason for stopping the NNRTI-regimen. For the purposes of the analysis the primary reason for switch was defined as *treatment failure, non-adherence* or *other* e.g. toxicity. If, both non-adherence and treatment failure were documented, the primary reason for switch was defined as non-adherence; if treatment failure and other reasons were documented the primary reasons was defined as treatment failure.

### Statistical Analysis

Modified Poisson regression with robust standard variance was used to estimate the association of exposures with viral suppression using the risk ratio [30]. This methodology was used, rather than logistic regression as the probability of the outcome was high and therefore the rare event assumption (odds and risk of an event are similar when the outcome is rare) did not hold true. By reporting risk ratios we avoided the possibility of the odds of an event being misinterpreted as risk and the strength of association being over-emphasized. A backwards stepwise approach was used whereby covariates associated with viral suppression (p≤0.2) in univariable analysis were considered for inclusion, and retained in the multivariable model if p≤0.2. Patients with missing outcome (died, left employment due to ill health, lost to follow-up or missing VL) were treated as failures; however patients who transferred out of clinic or left employment for reasons other than ill-health were excluded from the analysis. The Wald test was used to assess associations and, where appropriate, linearity and effect modification. Co-linearity was assessed by examining differences in standard errors between univariable and multivariable models.

Programme (community *vs*. workplace) was an effect modifier for multiple covariates (switch VL, transfers into programme on first-line ART, switch reason, age: p-value for interaction<0.05) therefore analyses are presented stratified by programme. Sensitivity analyses were performed by restricting analyses to patients who were ART-naive on initiating ART within the programme. Analyses were undertaken using STATA v11 (College Station, TX, USA).

### Ethics

This study was approved by the research ethics committees of the University of KwaZulu Natal, South Africa and the London School of Hygiene and Tropical Medicine, UK.

## Results

**Figure 1:**
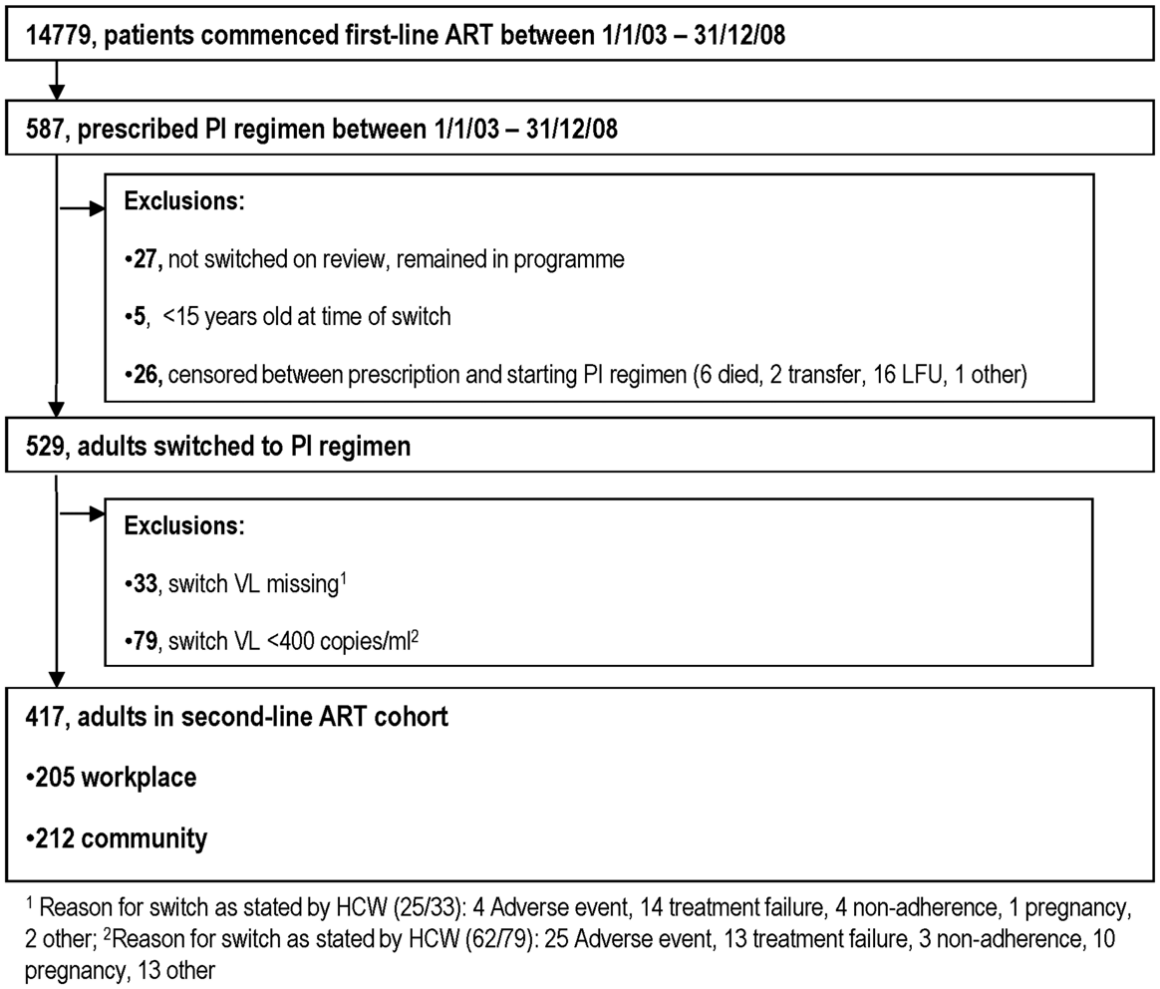
Study Flow diagram. Selection of adults for analysis, from a cohort of patients initiating first-line, NNRTI-based, ART between the 1^st^ January 2003 and 31^st^ December 2008.

Of 14779 patients who commenced first-line ART, 555 adults were prescribed second-line ART, of which 26 left the programme before ART was dispensed. In total 417/529 adults (205 workplace and 212 community programme) had a documented VL ≥400 copies/ml at switch and were eligible for inclusion in the study (figure 1).

The characteristics of patients who switched to second-line ART are presented in table 1. Compared to the community, patients in the workplace were older, more likely to be male, commenced first-line ART at a higher CD4 count and less advanced clinical stage, were more likely to be ART-naive when initiating first-line ART in the programme (10.8% vs. 52.1%) and have a longer duration on first-line prior to switch. Non-adherence on first-line ART was reported in a higher proportion of patients in the workplace *vs.* community programme. In both programmes, of the 62 patients classified as non-adherent on first-line ART, 21% of patients self-reported non-adherence and 84% had ART modified or interrupted for non-adherence by healthcare workers. More patients in the workplace programme were prescribed a second-line regimen consistent with programme guidelines (90.7% vs. 59.0% in the community programme); however 87.7% of community patients did modify at least one of the NRTI backbone drugs in addition to receiving a bPI.

**Table 1:** Baseline characteristics of patients receiving second-line ART, according to programme.

		Workplace	Community
		N = 205 (N, %)	N = 212 (N, %)
	**Age, years (median, IQR)**	43 (37-49)	36 (31-42)
	**Sex, male**	188 (91.7)	82 (38.7)
***Start of first-line ART***			
	**WHO clinical stage III or IV,** N = 152/190	108 (71.0)	164 (86.3)
	**CD4 at start of first-line, cells/mm^3^ (median, IQR),** N = 195/193	166 (91-221)	122 (43-195)
	**Transfers into programme on ART,** N = 185/192	20 (10.8)	100 (52.1)
***On first-line ART***			
	**Duration on first-line pre-switch, days (median, IQR)**	695 (447-1019)	517 (310-754)
	**Reported non-adherence**	54 (26.3)	8 (3.8)
	**Viral suppression,<400 copies/ml,** N = 190/151	130 (68.4)	108 (71.5)
***At switch to second-line ART***			
	**Documented reason for switch,** N = 180/192		
	Treatment failure	147 (81.7)	160 (83.3)
	Non-adherence	14 (7.8)	1 (0.5)
	Other e.g. toxicity, pregnancy	19 (10.6)	31 (16.1)
	**Year of switch**		
	≤2005	43 (21.0)	11 (5.2)
	2006-2007	57 (27.8)	84 (39.6)
	2008	105 (51.2)	117 (55.2)
	**Number of new NRTIs in switch regimen**		
	None	14 (6.8)	26 (12.3)
	1	7 (3.4)	46 (21.7)
	≥2	184 (89.8)	140 (66.0)
	**Duration of viraemia at switch** 1 **,** N = 205/207	82 (40.0)	95 (45.9)
	<12 months		
	≥12 months	123 (60.0)	112 (54.1)
	*Duration of viraemia between viral suppression and switch^1a^, days (median, IQR), N = 129/108*	365 (173-538)	218 (115-394)
	*Duration of viraemia in ART-naïve patients without viral suppression^1b^, days (median, IQR), N = 60/43*	538 (330-766)	368 (114-544)
	**VL(log_10_) at switch (median, IQR)**	4.6 (4.1-5.1)	4.3 (3.7-4.6)
	**CD4 at switch, cells/mm^3^ (median, IQR)**	169 (97-235)	187 (95-270)

1 Duration of viraemia was defined as (a) Patients with viral suppression on first-line ART: date of first viral load >400 copies/ml following viral suppression to date of switch, N = 237 (57.5%)^1a^; (b) ART-naive patients with no viral suppression on first-line ART: date of commencing first-line ART to date of switch, N = 103 (25.0%)^1b^; (c) Patients with ART-experienced pre-programme who did not achieve viral suppression on first-line ART: assumed to be ≥12months, N = 72 (17.4%). Abbreviations: IQR, inter-quartile range; VL, viral load; NRTI, nucleoside reverse transcriptase inhibitor.

A longer median duration of viraemia was observed amongst patients in the workplace *vs.* community programme; 365 days (IQR 173-538) *vs.* 218 days (IQR 115-394) in patients with viral suppression on first-line ART. In both programmes there was a median of 3 detectable VLs prior to switch (range: workplace 1-13, community 1-10). At switch, compared to the community, patients in the workplace programme had a higher median log_10_ VL (4.6 [IQR 4.1-5.1] vs. 4.3 [IQR 3.7-4.6]) and a lower median CD4 count (169 cells/mm^3^ [IQR 97–235] *vs.* 187 [IQR 95–270]).

### Reasons for Switching

In both programmes treatment failure was the commonest documented reason for switching regimens (workplace 82.2% [148/180 patients with recorded reason] *vs.* community 83.8% [161/192]). Non-adherence was recorded as a reason for switch in 7.8% (n = 14) of the workplace *vs.* 0.5% (n = 1) of the community programme. 10.6% [19/180] of patients in the workplace *vs.* 16.1% [31/192] in the community had other reported reasons for switching e.g. toxicity, although all were viraemic at the time of switching.

The two VLs prior to switch were ≥1000 copies/ml in 80.6% (336/417) of patients switched to second-line ART; in 16.1% (n = 67) the VL at switch was ≥1000 copies/ml with the preceding measurement 400-999 copies/ml or missing; and in 3.3% (n = 14) the switch VL was 400-999 copies/ml with the preceding measurement ≥400 copies/ml or missing.

### Clinical outcomes on Second-Line Art

Outcomes stratified by programme are presented in Table 2. 73.7% (N = 179) of patients in the workplace and 84.4% (N = 151) in the community programme were alive and in care (p<0.01) at 15 months, with 48.3% (N = 98) *vs.* 72.0% (N = 152), respectively, having achieved viral suppression (p<0.01) by 15 months. Patients in both programmes had a median of 5 VLs following switch, with 87.3% (workplace) and 88.7% (community) with ≥1 measurement. Of the 250 patients who achieved viral suppression, 19.2% had no further VL measurements within the follow-up period. Of those with further measurements, 35.6% (26/73) of patients in the workplace *vs.* 13.2% (17/129) of those in the community experienced a subsequent episode of viral rebound to ≥400 copies/ml (p<0.01; median 3 measurements [range 2-8 workplace, 2-5 community]). At 12 months (+/−3 months), of the patients who were still in care, 46.8% (59/126) of workplace and 72.0% (116/161) of community programme had a VL<400 copies/ml.

**Table 2:** Outcomes at 15 months of second-line ART.

		Workplace	Community	
		N = 205 (N, %)	N = 212 (N, %)	p^a^
**Clinical outcomes at 15 months**				
	Alive and in care	151 (73.7)	179 (84.4)	<0.01
	Died^b^	12 (5.8)	12 (5.7)	-
	Lost to follow-up	29 (14.0)	15 (7.1)	-
	Transfer out	-	5 (2.4)	-
	Other e.g. left employment	13 (6.3)	1 (0.5)	-
**Non-adherence reported on second-line ART**		37 (17.9)	3 (1.4)	<0.01
**Change in CD4 count from switch to 12m following switch, range 9-15m (mean, 95% CI),** N = 127/162		+68 (40-95)	+127 (101-154)	<0.01
**VL<400 within 15m of regimen start, range 2wks-15m,** c N = 203/211		98 (48.3)	152 (72.0)	<0.01
**Viral rebound (≥400) following initial viral suppression,** d N = 73/129		26 (35.6)	17 (13.2)	<0.01

a Chi^2^ was used for comparison of proportions; paired t-test was used for comparison of mean CD4 count increase;

b cause of death was available for 19/24 patients: 12 "natural causes" not further specified, 3 pneumonia, 1 tuberculosis, 1 cryptococcal meningitis, 1 gastroenteritis, 1 cerebro-vascular accident;

c Patients with missing outcome who transferred out of programme or left employment for reasons other than ill-health were excluded from the analysis (N = 2 workplace, N = 1 community). All other patients with missing outcome were treated as failures (N = 11 workplace, N = 12 community);

d Patients with ≥1 VL measurement following initial viral suppression (VL<400) on second-line ART.

Patients in the workplace had a lower mean CD4 count increase at 12 months of second-line ART than those in the community programme (p<0.01). Non-adherence was reported in a higher proportion of patients in the workplace, compared to the community programme (17.9% [workplace] *vs.* 1.4% [community]). In both programmes, of the 40 patients classified as non-adherent on second-line ART, 19% were identified through patient self-report and 83% through healthcare workers modification or interruption of ART for non-adherence.

### Predictors of Viral Suppression on Second-Line Art

Unadjusted and adjusted analysis of variables associated with viral suppression in the workplace and community programme are summarised in tables 3 and 4. In adjusted analysis of the workplace programme, a lower log_10_ VL (adjusted risk ratio [aRR] 1.59 [95% CI: 1.09-2.34] for<4 *vs.* ≥5) and younger age (aRR 0.87 [95% CI: 0.79-0.95]/5 year increase) at switch were the strongest predictors of viral suppression. In addition, our data suggests an association between switch for non-adherence *vs.* treatment failure (aRR 0.45 [95% CI: 0.17-1.16]) and lack of viral suppression on second-line ART. While the association did not reach statistical significance, the effect size was large. Duration of viraemia was not associated with viral suppression on second-line ART.

**Table 3:** Predictors of early viral suppression (viral load<400 copies/ml) on second-line ART in the workplace programme.

		Viral suppression	Univariable, N = 203		Multivariable, N = 178	
		N/at risk (%)	RR (95% CI)	p^a^	aRR (95% CI)	p^a^
**Transfers into programme on ART**, N = 184						
	**Yes**	7/20 (35.0)	0.69 (0.37-1.28)			
	**No**	83/164 (50.6)	1	0.24		
**Viral suppression, first-line ART**, N = 189						
	**Yes**	66/130 (50.8)	1	0.53		
	**No**	27/59 (45.8)	0.90 (0.65-1.25)			
**Reported non-adherence, first-line ART**						
	**Yes**	23/54 (42.6)	0.85 (0.60-1.20)			
	**No**	75/149 (50.3)	1	0.35		
**Reason for switch**, N = 178						
	**Treatment failure**	76/145 (52.4)	1		1	
	**Other**	8/19 (42.1)	0.80 (0.46-1.39)	0.44	0.81 (0.49-1.34)	0.41
	**Non-adherence**	3/14 (21.4)	0.41 (0.15-1.13)	0.08	0.45 (0.17-1.16)	0.1
**Year of switch**						
	**≤2007**	55/100 (55.0)	1	0.06	1	0.12
	**2008**	43/103 (41.8)	0.76 (0.57-1.01)		0.79 (0.59-1.07)	
**Duration of viraemia**						
	**<12 months**	42/82 (51.2)	1.11 (0.83-1.47)			
	**≥12 months**	56/121 (46.3)	1	0.49		
**VL(log_10_) at switch**						
	**≥5**	22/58 (37.9)	1	<0.01	1	<0.01
	**4-4.99**	45/103 (43.7)	1.15 (0.77-1.71)		0.87 (0.58-1.33)	
	**<4**	31/42 (73.8)	1.95 (1.34-2.83)		1.59 (1.09-2.34)	
**CD4 at switch**						
	**<100**	22/54 (40.7)	1	0.17^b^		
	**100-199**	36/74 (48.7)	1.19 (0.80-1.78)			
	**≥200**	40/75 (53.3)	1.31 (0.89-1.93)			
**New NRTIs in switch regimen**						
	**≤1**	11/21 (52.4)	1.10 (0.71-1.69)			
	**≥2**	87/182 (47.8)	1	0.68		
**Age at switch, per 5 years increase**		98/203 (48.3)	0.86 (0.79-0.94)	0.01^b^	0.87 (0.79-0.95)	<0.01^b^
**Gender**						
	**Male**	87/186 (46.8)	1	0.10		
	**Female**	11/17 (64.7)	1.38 (0.94-2.03)			
**Reported non-adherence, second-line ART^c^**						
	**Yes**	12/36 (33.3)	0.47 (0.22-1.00)			
	**No**	86/167 (51.5)	1	0.05		

a Wald test; ^b^ test for linear trend with no evidence of departure from linearity (CD4, p = 0.84; Age, p = 0.47); ^c^ not included in the multivariable model as considered to be on the causal pathway between exposures at time of switch and viral suppression on second-line ART.

In adjusted analysis of the community programme, shorter duration, but not magnitude of viraemia, predicted viral suppression (<12 months aRR 1.22 [95% CI: 1.03–1.44] *vs.* ≥12 months). Patients who were transferred into the programme on ART, and those switched at a higher CD4 count were more likely to suppress following switch. Sensitivity analyses of both programmes, restricting to ART-naïve patients, resulted in similar models (data not presented).

**Table 4:** Predictors of early viral suppression (viral load<400 copies/ml) on second-line ART in the community programme.

		Viral suppression	Univariable, N = 211		Multivariable, N = 191	
		N/at risk (%)	RR (95% CI)	p^a^	aRR (95% CI)	p^a^
**Transfers into programme on ART**, N = 191						
	**Yes**	82/100 (82.0)	1.33 (1.10-1.61)		1.33 (1.11-1.61)	
	**No**	56/91 (61.5)	1	<0.01	1	<0.01
**Virological suppression, first-line ART**, N = 150						
	**Yes**	75/108 (69.4)	1	0.56		
	**No**	27/42 (64.3)	0.93 (0.71-1.20)			
**Reported non-adherence, first-line ART**						
	**Yes**	5/8 (62.5)	0.86 (0.50-1.49)			
	**No**	147/203 (72.4)	1	0.60		
**Reason for switch,^b^** N = 190						
	**Treatment failure**	114/159 (71.7)	1	0.77		
	**Other**	23/31 (74.2)	1.03 (0.82-1.30)			
**Year of switch**						
	**≤2007**	74/95 (77.9)	1	0.08		
	**2008**	78/116 (67.2)	0.86 (0.73-1.02)			
**Duration of viraemia**, N = 206						
	**<12 months**	72/94 (76.6)	1.11 (0.94-1.32)		1.22 (1.03-1.44)	
	**≥12 months**	77/112 (68.7)	1	0.21	1	0.02
**VL(log10) at switch**						
	**≥5**	20/29 (69.0)	1	0.59		
	**4-4.99**	80/114 (70.2)	1.02 (0.77-1.34)			
	**<4**	52/68 (76.5)	1.11 (0.84-1.46)			
**CD4 at switch**						
	**<100**	32/54 (59.3)	1	0.01	1	0.02
	**100-199**	56/67 (83.6)	1.41 (1.10-1.80)		1.37 (1.05-1.78)	
	**≥200**	64/90 (71.1)	1.20 (0.93-1.55)		1.16 (0.88-1.52)	
**New NRTIs in switch regimen**						
	**≤1**	54/72 (75.0)	1.06 (0.90-1.26)			
	**≥2**	98/139 (70.5)	1	0.48		
**Age at switch**						
	**<35**	65/90 (72.22)	1.07 (0.83-1.38)			
	**35-44**	62/84 (73.81)	1.09 (0.84-1.41)			
	**≥45**	25/37 (67.57)	1	0.80		
**Gender**						
	**Male**	60/82 (73.2)	1	0.77		
	**Female**	92/129 (71.3)	0.97 (0.82-1.16)			
**Reported non-adherence, second-line ART ^c^**						
	**Yes**	1/3 (33.33)	0.19 (0.02-2.12)			
	**No**	151/208 (72.60)	1	0.16		

a Wald test; ^b^ only one patient switched regimens for non-adherence in the community programme. This patient has therefore been excluded as it would not be possible to assess a potential association); ^c^ not included in the multivariable model as considered to be on the causal pathway between exposures at time of switch and viral suppression on second-line ART.

## Discussion

We have demonstrated, in a community ART programme delivered by a network of private general practitioners and non-government organisations, outcomes on second-line ART, both in terms of remaining in programme and achieving viral suppression, which are comparable to those reported from academic referral clinics [25]-[26]. In contrast, in the workplace programme, over a quarter of patients were no longer alive and in care by 15 months of second-line ART and less than half achieved viral suppression.

The differences in outcomes by programme are of concern and are surprising given that both programmes use similar switch guidelines. We hypothesise that variations in healthcare workers’ switching practices, together with both individual and programme factors, may explain these outcomes.

Although guidelines were similar for both programmes, differences in switching practices were evident; patients in the workplace were switched at a more advanced stage of immune-suppression with a higher log_10_ VL, lower CD4 count and longer duration of viraemia. This was not explained by baseline characteristics at initiation of first-line ART; patients in the community initiated ART at a more advanced stage of HIV than in the workplace programme. Although prolonged viraemia in the presence of drug pressure is associated with NRTI cross-resistance [22]-[24], we do not believe that resistance is an adequate explanation for the different virological outcomes observed between programmes. Firstly, in settings without prior exposure to boosted PIs, given the potency of these drugs, high rates of early viral suppression are expected even in patients with extensive thymidine analogue mutations [26], [31]-[34]. Secondly, although the duration of viraemia was shorter in the community programme, over half of the patients were viraemic for more than 12 months and are thus likely to also have resistance.

We hypothesise that differences in healthcare workers’ implementation of switch guidelines, and the extent to which non-adherence is excluded prior to switching regimens, will influence early virological outcomes on second-line ART. Current guidelines give little indication of how best to manage patients who are believed to be non-adherent and who continue to experience virological failure despite intensified adherence interventions. Perceived non-adherence has been shown to influence healthcare workers decisions regarding ART prescribing [35].

In the community programme a longer duration of viraemia and lower CD4 count at switch predicted failure to achieve viral suppression on second-line ART. A longer duration of viraemia may be acting as a marker of non-adherence on first-line ART (albeit that drug resistance mutations could be accumulating) with these patients requiring a longer period to address adherence issues before the regimen is switched. However, patients considered adherent are switched quickly; this is consistent with these individuals being more likely to achieve viral suppression.

In the workplace programme, switch VL<10000 was one of the strongest predictors of viral suppression. While patients switched at higher VLs may take longer to suppress, the great majority should have achieved viral suppression by 15 months. An alternative explanation is that a high VL reflects non-adherence [12], [36]. Undisclosed non-adherence can result in healthcare worker misclassification of the aetiology of viraemia. Healthcare worker documented reason for switch will therefore only partially adjust for non-adherence. In the workplace cohort 14 patients had their NNRTI-regimen stopped for non-adherence and were switched to second-line ART; in multivariable analysis there is a suggestion that these patients were less likely to achieve viral suppression than those switched for treatment failure alone. This finding is consistent with results from other studies which report low rates of viral suppression amongst patients switched to second-line ART in the presence of wild-type virus [26], [37]-[39].

Not only were patients in the workplace programme less likely to achieve viral suppression on second-line ART, they were also more likely to be lost to the programme and to experience viral rebound following initial suppression. On univariable analysis of data from the workplace programme non-adherence on second-line ART was associated with failure to achieve viral suppression. In the community programme the analysis was underpowered to assess an association due to low levels of reported non-adherence. While it is possible that failure to achieve viral suppression and viral rebound is due to emergence of early PI resistance we feel this is unlikely; other studies in resource-limited settings indicate that early second-line failure is more likely to be due to non-adherence, with PI mutations rarely seen and low PI concentrations reported [40]-[42]. We believe that these early viral rebounds are secondary to failure to sustain improved adherence behaviour which resulted from adherence interventions implemented at the time of switch. This could be due to contextual factors influencing patients’ adherence behaviour or failure of the health-care system to adequately support patients at high risk of non-adherence.

Although predictors of viral suppression differed between programmes, overall the findings are consistent with other studies; duration and magnitude of viraemia [37], [43], CD4 count at switch [38], [43], recent calendar year [44], older age [38], adherence [25], [31], [37]-[38] and prior-ART [25] have all been shown to be associated with second-line virological outcomes.

Other studies in this setting have also highlighted differences in switching rates and first- and second-line outcomes by site [40], [45]-[49]; Pujades-Rodriguez *et al.* report differences in switching rates between urban and rural sites, while others have found clinic type to be associated with second-line virological failure [40], [45]. The underlying reasons are multi-factorial with patient, health-system and community factors contributing [47]-[51].

The observed differences in programme outcomes may in part be due to the different patient populations, both in terms of individuals and the community, and the healthcare systems. The workplace population was older, predominantly male, comprised largely of migrants living in close proximity to their site of work, with access to only one major healthcare provider. In contrast patients accessing the community programme were younger, mostly female, and while potentially migrants, had a choice of healthcare provider.

Higher levels of non-adherence were reported amongst patients on first- and second-line ART in the workplace, compared to the community programme. While differences in non-adherence between programmes may be due to differences in reporting, studies conducted in this workplace setting have demonstrated multiple barriers to maintaining adherence including lack of social support, uncertainty about ART’s health benefits, belief in traditional medicine and patient-provider language barriers [48], [52]. While not unique to this setting [53], these barriers may be more prevalent amongst patients in a workplace as compared to a community setting. Indeed higher levels of non-adherence have been reported amongst patients enrolled in one of the workplace clinics *vs.* a government public clinic [48]. Patients within the workplace were older than those in the community programme and within the workplace programme older patients were less likely to achieve viral suppression. In many studies older age is associated with better adherence and superior outcomes [54]-[59], however this association may not be generalisable to the workplace setting where older age is a perceived barrier to adherence [52]. In resource-limited settings male gender has been associated with later initiation of ART [60], defaulting [59], [61]-[62], non-adherence [63]-[64] and mortality [64]-[68]. The association between gender and viral suppression varies; some studies report an association between female gender and first-line viral suppression [69]-[70] others between male gender and second-line viral suppression [71], These studies are from different settings, from urban townships to rural programmes; while biological characteristics may contribute, the association with gender is likely to be influenced by societal determinants specific to each setting. Within the community programme we did not find any association between gender and second-line virological suppression, and we were unable to assess the role of gender within the workplace as the majority of patients were male. Finally approximately half of the patients on second-line ART in the community programme were transfers into care, in some cases these were patients who self-funded first-line ART but could not afford more expensive second-line regimens (personal communication, S Charalambous). This was the strongest predictor of viral suppression in the community cohort. We believe patients who transfer between healthcare providers are likely to be highly motivated individuals [25], [48]. Fox *et al.* report similar findings; patients switched after only one VL, who were considered to be transfers into care on ART, were more likely to achieve viral suppression on second-line ART [25].

This study included large patient numbers across multiple sites. Extensive efforts were made to limit measurement bias and reduce effects of missing data by reviewing clinic notes, verification of switch date by cross-checking with pharmacy data, and ascertainment of deaths through multiple sources (linkage to national death register, company employment records and hospital death register).

There are limitations to our analysis. It was based on routinely collected programme data; clinic- and contextual level covariates e.g. clinic staffing levels or patients’ migrant status which could influence outcomes were not available. Due to lack of resistance data, incomplete programme reporting of non-adherence and inaccuracy of self-report as a measure of adherence, we were unable to fully explore the respective roles of resistance and adherence in early second-line virological outcomes. In addition, as programme acted as an effect modifier for multiple covariates, our analysis was stratified by programme. We were therefore unable to quantify the effect of programme (workplace vs. community) adjusted for potential confounders. Other limitations include that, for pragmatic purposes, our definition of duration of viraemia was a composite measure; for patients who did not achieve viral suppression on first-line ART, duration of viraemia was dependant on knowledge of pre-programme ART exposure and duration in programme. While our definition was subject to measurement error we do not believe it has resulted in bias; there was no evidence of co-linearity between ART exposure and duration of viraemia in the community programme multivariable model, and using an alternative definition based on duration of observed viraemia while in programme, similar associations were found. Also, with no difference in frequency of virological monitoring between programmes it is unlikely that detection bias would explain the differences in duration of viraemia, nor indeed virological outcomes. Finally in both second-line cohorts, the majority of patients were cared for by four clinics and the results may therefore be biased towards practices in these larger clinics. It is possible that the programmatic differences we have described in this study relate more to the individual clinics, rather than the programmes themselves. In a larger study looking at predictors of switching to second-line ART we found switching varied markedly by clinic. Programme, however was not associated with switching, nor did it account for clinic-level clustering [49]. As the majority of clinics contributed only one to two patients to this second-line analysis, clustering by clinic was not adjusted for.

### Conclusion

The results from this study reflect the real-life dilemmas encountered in managing virological failure and switching to second-line ART in a resource-constrained setting. We demonstrate that it is possible to achieve high levels of viral suppression on second-line ART in multi-site programmes; however this is not true of all settings with both individual- and programme-level factors influencing outcomes. Despite similar guidelines, switching practices differed between programmes. With no access to resistance tests and imperfect adherence assessment tools, deciding who is failing therapy and might benefit from switching is difficult. The factors driving sub-optimal adherence, particularly in the workplace programme, need addressed and strategies to support switch decisions, such as targeted resistance tests, which may be cost-neutral, warrant further investigation [72].
